# Pathological characteristics of axons and proteome patterns in midbrain dopaminergic neurodegeneration induced by WDR45-deficiency

**DOI:** 10.21203/rs.3.rs-2901370/v1

**Published:** 2023-05-18

**Authors:** Weidong Le, Panpan Wang, Murad Al-Nusaif, Jun Zhang, Huijia Yang, Yuting Yang, Kunhyok Kim, Song Li, Cong Liu, Huaibin Cai

**Affiliations:** The First Affiliated Hospital Of Dalian Medical University; First Affiliated Hospital of Dalian Medical University; First Affiliated Hospital of Dalian Medical University; First Affiliated Hospital of Dalian Medical University; First Affiliated Hospital of Dalian Medical University; First Affiliated Hospital of Dalian Medical University; First Affiliated Hospital of Dalian Medical University; First Affiliated Hospital of Dalian Medical University; Shanghai Institute of Organic Chemistry; NIH: National Institutes of Health

**Keywords:** WDR45, neurodegeneration, axonal degeneration, tubular ER, autophagy, phospholipid metabolism

## Abstract

**Background:**

Although *WD repeats domain 45 (WDR45)* mutations have been linked to *β*-propeller protein-associated neurodegeneration (BPAN), the precise molecular and cellular mechanisms behind this disease remain elusive. This study aims to shed light on the effects of WDR45-deficiency on neurodegeneration, specifically axonal degeneration, within the midbrain dopaminergic (DAergic) system. By examining pathological and molecular alterations, we hope to better understand the disease process.

**Methods:**

To investigate the effects of WDR45 dysfunction on mouse behaviors and DAergic neurons, we developed a mouse model in which WDR45 was conditionally knocked out in midbrain DAergic neurons (*WDR45*^*cKO*^). Through a longitudinal study, we assessed alterations in mouse behavior using open field, rotarod, Y-maze, and 3-chamber social approach tests. To examine the pathological changes in DAergic neuron soma and axons, we utilized a combination of immunofluorescence staining and transmission electron microscopy. Additionally, we performed proteomic analyses of the striatum to identify the molecules and processes involved in striatal pathology.

**Results:**

Our study of *WDR45*^*cKO*^ mice revealed a range of deficits, including impaired motor function, emotional instability, and memory loss, coinciding with the profound loss of midbrain DAergic neurons. Prior to neuronal loss, we observed massive axonal enlargements in both the dorsal and ventral striatum. These enlargements were characterized by the accumulation of extensively fragmented tubular endoplasmic reticulum (ER), a hallmark of axonal degeneration. Additionally, we found that *WDR45*^*cKO*^ mice exhibited disrupted autophagic flux. Proteomic analysis of the striatum in these mice showed that many differentially expressed proteins (DEPs) were enriched in amino acid, lipid, and tricarboxylic acid metabolisms. Of note, we observed significant alterations in the expression of genes encoding DEPs that regulate phospholipids catabolic and biosynthetic processes, such as lysophosphatidylcholine acyltransferase 1, ethanolamine-phosphate phospho-lyase, and abhydrolase domain containing 4, N-acyl phospholipase B. These findings suggest a possible link between phospholipid metabolism and striatal axon degeneration.

**Conclusions:**

In this study, we have uncovered the molecular mechanisms underlying the contribution of WDR45-deficiency to axonal degeneration, revealing intricate relationships between tubular ER dysfunction, phospholipid metabolism, BPAN and other neurodegenerative diseases. These findings significantly advance our understanding of the fundamental molecular mechanisms driving neurodegeneration and may provide a foundation for developing novel, mechanistically-based therapeutic interventions.

## Background

*Tryptophan-aspartic acid (WD) repeat domain 45 (WDR45)*, encoding a superfamily of proteins characterized by repeating units with a conserved core of approximately 40 amino acids, is located on the X-chromosome (1). WD-repeat proteins have a highly symmetrical β-propeller tertiary structure that enables them to regulate the assembly of multiprotein complexes by providing a stable anchoring platform (2). Based on this structural property, it seems that the WDR45 protein plays key roles in many biological processes, including autophagy, an autophagosome-lysosome-mediated degradation system (3), transduction, and vesicular trafficking (4, 5). De novo mutations in the *WDR45* gene were recently identified in β-propeller protein-associated neurodegeneration (BPAN) disease (6, 7). BPAN is characterized as a subtype of neurodegeneration with brain iron accumulation, and clinically, patients with *WDR45* mutations have a biphasic disease course that begins with global developmental delay in infancy or early childhood, and subsequent progressive cognitive decline, dementia, dystonia, and Parkinsonism in adolescence or early adulthood (8–10). Moreover, brain imaging from BPAN patients with mutated *WDR45* displays generalized brain atrophy and bilateral mineralization of the substantia nigra (SN) and globus pallidus (8, 11). In addition to BPAN disorder, *WDR45* mutations have been linked to pancreatic cancer, kidney cancer, and Rett syndrome (12, 13). Rett syndrome is a neurodevelopmental disorder characterized by the loss of purposeful hand skills, language regression, stereotypic hand movements, and gait abnormalities. Some BPAN patients have features similar to Rett syndrome (13, 14).

The endoplasmic reticulum (ER) is an interconnected network of tubular and planar membranes that supports synthesizing and exporting proteins, carbohydrates, and lipids (15). ER is also the primary site for phospholipid synthesis, particularly phosphatidylcholine (PC) synthesis, and accounts for more than 60% of phospholipid mass (16). The phospholipid content and composition of the ER are dynamic for the regulated export to other membrane organelles and secretion of lipids to the extracellular environment. Lipid biosynthesis needs to match these export demands to maintain ER integrity and the membrane requirements of distal organelles. The extended tubular and planar ER network extends to all cell regions, where it interfaces at membrane contact sites with the plasma membrane, mitochondria, Golgi apparatus, lipid transfer, and calcium homeostasis. The regulation of lipid metabolic enzymes could induce membrane deformation because many of them, like phosphate cytidylyltransferase 1A, choline (17), and phospholipase A2 group IVA, contain membrane curvature-inducing domains and phospholipid composition (18).

Axonal ER comprises tubular ER and forms a physically continuous network of interconnected tubular structures, participating in axonal morphology, transport, and material metabolism, suggesting its potential role in neurodegeneration (19). The tubular ER structure is determined by ER-shaping proteins, such as reticulons (RTNs), receptor accessory proteins (REEPs), and the atlastins (ATLs) family.

Mutations in these ER-shaping proteins can cause hereditary spastic paraplegia (19). The accumulation of tubular ER is primarily specific to axons (20). Recent studies have revealed the physiological roles of tubular ER in neurodegenerative disorders (19, 21, 22). Autophagy gene 5 (ATG5) deletion leads to tubular ER accumulation in axons of hippocampal neurons (20), demonstrating that autophagy participates in the axonal tubular ER degradation.

The tubular ER dynamics are closely linked to phospholipid synthesis. Cytidine diphosphate-triacylglycerol (CDP-DAG) synthase 1, a crucial enzyme of PC synthesis through the CDP-DAG pathway, is uniformly localized to the tubular ER and nuclear envelope. Phosphatidylinositol synthase (PIS), the rate-limiting enzyme for phosphatidylinositol synthesis, also localizes to the tubular ER network. Rab10, an ER-specific Rab GTPase, regulates ER tubule dynamics and tubular ER morphology by controlling ER tubule extension and fusion at the leading edge of ER dynamics. Further study suggests that these dynamics could be coupled to phospholipid synthesis since the Rab10 domain is highly enriched with at least two ER enzymes that regulate phospholipid synthesis, PIS, and choline/ethanolamine phosphotransferase 1 (23).

Using a line of newly developed WDR45 conditional knockout (cKO) mice, our study shows a correlation between axonal degeneration and the accumulation of aberrant tubular ER in WDR45-deficient midbrain DAergic neuron. These findings suggest that the dysfunction of autophagy and alterations in lipid metabolism, particularly phospholipid metabolism, may play a role in striatal axonal degeneration. By uncovering these connections, our study provides new insights into the molecular mechanisms underlying WDR45-deficiency-induced neurodegeneration and highlights potential targets for developing therapeutic interventions to prevent or reverse this process.

## Materials and methods

### Generation of the conditional knockout *WDR45* Mouse model

Heterozygous *WDR45*^*Flox/wt*^ mice were generated by ViewSolid Biotech Co., Ltd. (Beijing, China). Briefly, CRISPR/Cas9 technology replaced the genomic DNA fragment from intron 2 and 4 of the WDR45 gene with a donor DNA fragment containing LoxP-flanked exon 2 to 4 WDR45. Based on Cas9/gRNA activity screen and target location, high-activity gRNAs (target DNA sequence: GCACAAACACCAAGCATGGGG; ACACAGTGCTATTGGGGCTGG) were selected for microinjection into C57BL/6J fertilized eggs to produce conditional gene knockout mice.

To achieve a mouse model that conditionally knocked out *WDR45* in the DAergic system, we bred *DAT*^*CreERT2*^ mice carrying inducible Cre recombinase under the DAT promoter with heterozygous *WDR45*^*Flox/wt*^ mice to obtain *WDR45*^*Flox/Flox*^*/DAT*^*CreERT2*^ mice. The *DAT*^*CreERT*2^ mouse was kindly gifted by the Günther Schütz group and was generated by recombining a construct containing a modified Cre recombinase fused to a modified ligand-binding domain of the estrogen receptor into a bacterial artificial chromosome containing the gene encoding DAT.

All mice were maintained under SPF conditions (temperature, 22 ± 2 °C; air exchange per 20 minutes; 12 h/12 h light/dark cycle with the light on at 6:00 AM) with free access to food and water. Animal care and procedures were carried out per the Laboratory Animal Care Guidelines approved by the Institutional Animal Care Committee at Dalian Medical University. The protocol was approved by the Institutional Animal Care Committee at Dalian Medical University.

To achieve the conditional knockout of WDR45 in the mature DAergic system, TAM (T-5648; Sigma‒ Aldrich) was employed to treat mice. TAM has dissolved in a corn oil/ethanol (S-5007; Sigma‒Aldrich) mixture with a ratio of 10:1 at a final concentration of 10 mg/mL. A fresh mixture was prepared by shaking overnight to dissolve TAM completely at 4 °C and then stored at −20 °C for 2 weeks. Eight-week-old *WDR45*^*Flox/Flox*^ *(WDR45*^*cWT*^*) and WDR45*^*Flox/Flox*^*/DAT*^*CreERT2*^ (*WDR45*^*cKO*^) mice were both injected intraperitoneally with 1 mg TAM twice daily (total 2 mg/day, approximately 25 mg/kg body weight) for 5 consecutive days. Behavioral tests were performed at the ages of 6–8 months, 11–13 months, and 17–19 months. *WDR45*^*cWT*^ and *WDR45*^*cKO*^ mice were sacrificed at the scheduled time (Fig. S1a).

*DAT*^*CreERT2*^ transgenic mice were identified by PCR screening (2 × EasyTaq PCR SuperMix, Transgene Biotech) of tail DNA using an antisense primer, CAG ACC AGG CCA GGT ATC TCT, and a sense primer, AGA ACC TGA TGG ACA TGT TCA GG, of which the transgene band size is 700 bp. Floxed WDR45 knock-in mice were identified using CCACAGTAAGGCACAGTT and GTACAGACCAGGCAAGTG. The PCR product size of the wild-type allele was 179 bp, and the knock-in Flox allele was 213 bp.

### Behavioral tests

#### 1. Locomotor activity

To examine their locomotor activity, *WDR45*^*cWT*^ and *WDR45*^*cKO*^ mice were placed in a locomotor activity monitor (25 × 25 × 30 cm, Med Associates Inc., St. Albans, USA) equipped with a computer-controlled photocell. The activity was automatically tracked and recorded for 10 minutes for the total distance traveled and stereotypic time. The assessment was conducted on the scheduled date between 13:00 and 16:00 at the ages of 6–8 months, 11–13 months, and 17–19 months.

#### 2. Rotarod test

As described previously, mice were trained on the IITC Rotarod (IITC Life Science, Woodland Hills, CA) at 5 r/minutes, twice per day (at 1-hour intervals) for 3 consecutive days, and then on the fourth day, they were tested on the rotating rod with speed auto accelerating from 4 to 40 r/minutes over a period of 5 minutes. The time spent on the rotating rod for each mouse was recorded across three trials at 1-hour intervals. The behavioral assessment was performed at ages 6–8 months, 11–13 months, and 17–19 months.

#### 3. Y-maze test

The Y-maze test apparatus (Beijing Zhongshidichuang Science and Technology Development Col., Ltd, Beijing, China) was implemented on a white background with three arms (labeled a, b, and c arms) that extended from a central platform at a 120° angle. Each mouse was placed in the center and allowed to freely explore the maze for 6 minutes. The sequence and the total number of arms that the mouse entered were recorded using the observer. An arm entry was successful when the mouse’s whole body was within the arm.

#### 4. Three-chamber social approach test

The three-chamber social approach test was performed as described previously (4). Briefly, a conspecific mouse of the same sex was placed in a wire-framed steel cage within either the left or right chamber (named novel, the left steel named other), and the subject mouse was allowed to move freely among the three chambers for 5 minutes. A second novel mouse (matched for age and sex) was placed in the remaining wired framed steel cage (named novel, the previous one named familiar), and the subject mouse could move freely for an additional 5 minutes. The cumulative exploration time of the mouse to enter each zone was measured.

### Immunofluorescence (IFC) staining

Mice were anesthetized with ketamine and perfused transcardially with 40 mL PBS and then 60 mL 4% paraformaldehyde (PFA). After dehydrating 30% sucrose for 72 hours, the brain tissues were cut into 40 μm coronal sections using a Leica cryostat (CM-1950S, Leica, Germany). The slides were incubated with IFC blocking buffer (10% normal goat serum, 1% bovine serum albumin, 0.3% Triton X-100, PBS solution) for 2 hours at room temperature and were then incubated with the primary antibodies overnight at 4 °C (a complete list of primary antibodies information in Table 1). For Ub staining, the sections were subjected to antigen repair using citrate buffer (pH 6.0). The stained sections were visualized and photographed directly with a laser scanning confocal microscope (A1 confocal, Nikon Instruments (Shanghai) Co., Ltd). The paired images in the figures were collected at the same gain and offset settings. TH-positive cells in the SNc and VTA were calculated in every three sections from bregma −2.80 to −3.64 mm, and the data were collected from 9 slices per animal. The outline of the SNc and VTA was determined according to anatomical landmarks. The analysis of IFC staining on the number of the puncta, axon density, and the mean number of enlarged axon terminals was quantified using ImageJ software.

### Quantitative Real-Time PCR (qRT-PCR)

Mice were decapitated, and the striatum was isolated on ice. Total RNA was extracted using TRIzol reagent (Invitrogen, Carlsbad, CA, United States), and reverse transcription was performed according to the manufacturer’s instructions (638315, Clontech Laboratories, Inc., A Takara Bio Company, United States). qRT-PCR was performed to determine the expression levels of lipid metabolism-related genes using a proper qRT-PCR kit (a complete list of qRT-PCR primers information in Table 2).

### Transmission electron microscope (TEM) Analysis

The midbrain SN and striatum were collected rapidly on ice within 3 min into a fixative solution containing 2.5% glutaraldehyde (Servicebio, Wuhan, China) for 2 hours fixtion at room temperature, followed by transfer to 4 degrees for storage. The tissues were washed three times in PBS before postfixing in 1% osmium acid (diluted with 0.1 M PBS solution) at room temperature for 2 hours and were successively dehydrated. After embedding steps, tissues were cut into 80 nm sections using a Leica ultrathin microtome (Leica UC7, Leica, Germany) and stained with 2% uranyl acetate saturated alcohol and lead citrate solution. The stained sections were imaged using TEM (HITACHI, HT7700).

### Proteomic Analysis

#### 1. Sample preparation

The striatal samples were ground into cell powder with liquid nitrogen before being transferred to a 5-mL centrifuge tube. 4 mL lysis buffer (8 M urea, 1% protease inhibitor cocktail) was added to the cell powder, then sonicated three times on ice using a high-intensity ultrasonic processor (Scientz). The remaining debris was removed by centrifugation at 12,000 g at 4 °C for 10 min. Collecting the supernatant and the protein concentration were determined with a BCA kit according to the manufacturer’s instructions. For digestion, the protein solution was reduced with 5 mM dithiothreitol for 30 min at 56 °C and alkylated with 11 mM iodoacetamide for 15 min at room temperature in darkness. The protein sample was then diluted by adding 100 mM TEAB to urea concentration less than 2 M. Trypsin was added at 1:50 trypsin-to-protein mass ratio for the first digestion overnight and at 1:100 trypsin-to-protein mass ratio for a second 4 h-digestion. The peptides were desalted by the C18 SPE column.

#### 2. LC-MS/MS-based proteomic analysis

The tryptic peptides were dissolved in solvent A (0.1% formic acid, 2% acetonitrile/in water) and directly loaded onto a reversed-phase analytical column (25-cm length, 75/100 μm i.d.). Peptides were separated with a gradient from 6% to 24% solvent B (0.1% formic acid in acetonitrile) over 70 min, 24% to 35% in 14 min, and climbing to 80% in 3 min, then holding at 80% for the last 3 min at a constant flow rate of 450 nL/min on a nanoElute UHPLC system (Bruker Daltonics). The peptides were subjected to a capillary source followed by the timsTOF Pro (Bruker Daltonics) mass spectrometry. The electrospray voltage applied was 1.60 kV. Precursors and fragments were analyzed at the TOF detector, with an MS/MS scan range from 100 to 1700 m/z. The timsTOF Pro was operated in parallel accumulation serial fragmentation (PASEF) mode. Precursors with charge state 0 to 5 were selected for fragmentation, and 10 PASEF-MS/MS scans were acquired per cycle. The dynamic exclusion was set to 30 s. The MS/MS data were processed using MaxQuant search engine (v.1.6.15.0). Tandem mass spectra were searched against the human SwissProt database (20422 entries) concatenated with the reverse decoy database. Trypsin/P was specified as a cleavage enzyme allowing up to 2 missing cleavages. The mass tolerance for precursor ions was set as 20 ppm in the first search, and 5 ppm in the main search, and the mass tolerance for fragment ions was set as 0.02 Da. Carbamidomethyl on Cys was specified as a fixed modification, and acetylation on protein N-terminal and oxidation on Met were specified as variable modifications. FDR was adjusted to < 1%.

#### 3. Bioinformatics analysis for proteome

GO analysis mainly includes three aspects: 1. Cellular component: Refers to the specific component of the cell. 2. Molecular function: Mainly describe the chemical activity of the molecule, such as the catalytic activity or binding activity at the molecular level. 3. Biological process: a series of elements in the body that execute a specific function in order, called the biological process. GO annotation is to annotate and analyze the identified proteins with eggnog-mapper software (v2.0). The software is based on the EggNOG database. The latest version is the 5th edition, covering 5,090 organisms (477 eukaryotes, 4445 representative bacteria, and 168 archaebacteria) and 2502 virus genome-wide coding protein sequences. Extracting the GO ID from the results of each protein note and then classifying the protein according to cellular component, molecular function, and biological process. Fisher’s exact test is used to analyze the significance of GO enrichment of differentially expressed proteins (using the identified protein as the background), and *p* < 0.05 is considered significant.

### Statistical Analysis

Data are expressed as the means ± SEMs and were analyzed using GraphPad Prism software (version 9.0). Two-way ANOVA followed by Sidak’s multiple comparisons test was used for analyses across multiple groups, with Student’s t-test used to determine significant differences between the two groups. *p <* 0.05 is considered significant. All experiments were repeated at least three times, and pilot experiments estimated sample sizes.

## Results

### Progressive midbrain DAergic neuronal loss in the SN of WDR45 ^cKO^ mice

To create a mouse model with selective deletion of *WDR45* in the midbrain DAergic neurons, we used a TAM-inducible CreERT2/loxp gene-targeting system (Fig. S1a). We generated homozygous WDR45-floxed mice without or with the CreERT2 gene (*WDR45*^*cWT*^ and *WDR45*^*cKO*^, respectively) and confirmed their genotype using conventional PCR analysis (Fig. S1b). When the mice reached 8 weeks of age, we administered intraperitoneal injections of TAM to both *WDR45*^*cWT*^ and *WDR45*^*cKO*^ mice. Tissues were collected 4 months after TAM administration (when the mice were 6 months old), and we used immunofluorescence (IFC) staining to detect WDR45 protein expression in the tyrosine hydroxylase (TH)-labeled DAergic neurons. The results showed a marked decrease in WDR45 expression in the DAergic neurons of *WDR45*^*cKO*^ mice (Fig. S1c), indicating successful deletion of WDR45 in these neurons.

*WDR45* ^*cWT*^ and *WDR45*^*cKO*^ mice were subjected to behavioral tests at different ages, including 6–8 months (young), 11–13 months (middle-aged), and 17–19 months (aged). The results showed that the aged *WDR45*^*cKO*^ mice had a significant motor impairment, as indicated by a decrease in the total distance traveled in the open-field test (Fig. S2a) and a notable reduction in stereotypic counts (Fig. S2b), suggesting increased vulnerability to motor activity impairments with aging. However, no abnormalities were observed in the rotarod test (Fig. S2c). In addition to the locomotion deficits, the aged *WDR45*^*cKO*^ mice also showed poor immediate spatial working memory performance, as evidenced by a decreased spontaneous alteration proportion in the Y maze test (Fig. S2d). Furthermore, the 3-chamber social performance test revealed that the aged *WDR45*^*cKO*^ mice displayed a significant decrease in social behavior (Fig. S2e-h), suggesting that WDR45 dysfunction in midbrain DAergic neurons may lead to depression-like behavior in aging mice.

**Figure 1:**
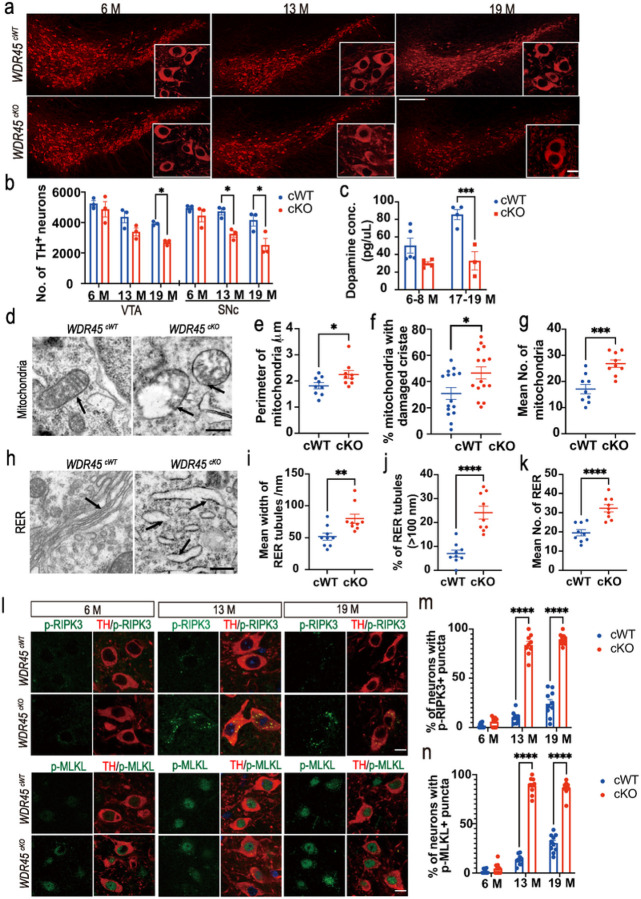
DAergic neuronal loss in the SN. a IFC staining was performed using an antibody against TH (red) in midbrains from young (6–8 months old), middle-aged (11–13 months old), and aged (17–19 months old) *WDR45* ^*cWT*^ and *WDR45*^*cKO*^ mice. Scale bar, 250 μm. Scale bar for high-magnification images, 10 μm. b Quantifying TH-positive neurons in the VTA and SNc of *WDR45*^*cWT*^ and *WDR45*^*cKO*^ mice (N=3 mice per genotype). c The dopamine concentration in the SN was detected by high-performance liquid chromatography (N=3–5 mice per genotype). d Representative TEM images of observed mitochondria in aged *WDR45*^*cWT*^ mice and *WDR45*^*cKO*^ mice. Scale bar, 500 nm. e Quantification of the perimeter of mitochondria in DAergic neurons (N= 154 mitochondria collectively counted from 9 slices of 3 *WDR45*^*cWT*^ mice and 251 mitochondria from 9 slices of 3 *WDR45*^*cKO*^ mice). f The proportion of mitochondria with damaged cristae was quantified (N= 9 slices from 3 mice per genotype). g The mean number of mitochondria observed was collected from images (N=9 slices from 3 mice for each genotype). h Representative TEM images of observed RER. Scale bar, 500 nm. i The mean width of RER tubules is shown (N= 163 RER collectively counted from 9 slices of 3 *WDR45*^*cWT*^ mice and 288 RER from 9 slices of 3 *WDR45*^*cKO*^ mice). j The proportion of RER tubules (>100 nm) was quantified (N=9 slices from 3 mice per genotype). k The mean number of RER observed was collected from images (N=9 slices from 3 mice for each genotype). l Double-label immunofluorescence of p-RIPK3 (Thr 231/Ser232) or p-MLKL (phosphor S345) (green) with TH (red) in the DAergic neurons of young, middle-aged and aged *WDR45*^*cWT*^ mice and *WDR45*^*cKO*^ mice. Scale bar, 10 μm. m The proportion of TH-positive neurons with p-RIPK3 puncta was quantified. (N= 3 mice per genotype). n The proportion of TH-positive neurons with p-MLKL puncta was quantified. (N= 3 mice per genotype). Data were analyzed using two-way ANOVA followed by Sidak’s multiple comparisons tests (b, d, m, n) and Student’s t-test (e-g, i-k). Data are represented as the mean±SEM. *p < 0.05, ***p < 0.001, ****p < 0.0001.

To further investigate the survival of DAergic neurons, we performed IFC staining for TH, a classic marker of DAergic neurons. We analyzed the number of DAergic neurons in the substantia nigra pars compacta (SNc) and ventral tegmental area (VTA) of *WDR45*^*cWT*^ and *WDR45*^*cKO*^ mice at young, middle-aged, and aged stages. We observed a reduction in the number of DAergic neurons in the VTA and SNc of middle-aged *WDR45*^*cKO*^ mice compared to age-matched *WDR45*^*cWT*^ mice, and this loss was significantly exaggerated in the aged *WDR45*^*cKO*^ mice (Fig. 1a, b). Additionally, we found a significant decrease in the DA content in the SN of aged *WDR45*^*cKO*^ mice compared to that of aged *WDR45*^*cWT*^ mice (Fig. 1c).

We conducted further analysis to investigate the impact of WDR45-deficiency on DAergic neurons at the subcellular level. We used TEM analysis to assess mitochondrial morphology in aged *WDR45*^*cKO*^ mice. Our results showed the presence of vacuolized mitochondria in the soma of DAergic neurons of aged *WDR45*^*cKO*^ mice but not in age-matched *WDR45*^*cWT*^ mice (Fig. 1d). The perimeter of mitochondria was significantly increased in *WDR45*^*cKO*^ mice compared to *WDR45*^*cWT*^ mice, and more than 40% of the mitochondria cristae in the DAergic neurons of *WDR45*^*cKO*^ mice were broken or disappeared (Fig. 1e, f), indicating mitochondrial damage upon WDR45-deficiency in DAergic neurons. We also analyzed the rough endoplasmic reticulum (RER) structure and found a significant change in RER morphology in aged *WDR45*^*cKO*^ mice (Fig. 1h). The mean width of RER tubules was significantly expanded in the DAergic neurons of *WDR45*^*cKO*^ mice compared to that in *WDR45*^*cWT*^ mice (79.9 nm vs. 51.7 nm) (Fig. 1i). The proportion of RER tubules (> 100 nm) was increased from 6.4% in the DAergic neurons of aged *WDR45*^*cWT*^ mice to 23.9% in the *WDR45*^*cKO*^ mice (Fig. 1j), indicating that the DAergic neurons suffered from severe RER tubular expansion due to *WDR45* deletion. Moreover, we found that the numbers of RER and mitochondria detected in the TEM images were dramatically increased in the DAergic neurons of *WDR45*^*cKO*^ mice compared to *WDR45*^*cWT*^ mice (Fig. 1g, k). These findings suggest that *WDR45* deletion may inhibit the clearance or turnover of damaged organelles, accelerating the degeneration of DAergic neurons.

Given that the *WDR45*^*cKO*^ mice experience progressive loss of DAergic neurons during aging, we aimed to investigate the mechanisms underlying cell death. Necroptosis is a regulated form of necrosis and is considered a new mode of cell death. When necroptosis is induced, receptor-interacting protein kinase-3 (RIPK3) becomes activated through phosphorylation and then phosphorylated RIPK3 activates mixed lineage kinase-like (MLKL) through phosphorylation (24, 25). To determine if necroptosis was activated in the DAergic neurons of the *WDR45*^*cKO*^ mice, we assessed the presence of phospho-RIPK3 and phospho-MLKL puncta in neuronal cytoplasm using our previously established methods (26). Our data revealed a marked increase in the concentrated puncta of phospho-RIPK3 and phospho-MLKL in the cytoplasm of middle-aged and aged *WDR45*^*cKO*^ mice (Fig. 1l, m, n). These results indicate that necroptosis was activated in the DAergic neurons, leading to progressive DAergic neuronal death in the *WDR45*^*cKO*^ mice during aging.

### Axonal degeneration in the striatumof WDR45 ^cKO^ mice

**Figure 2:**
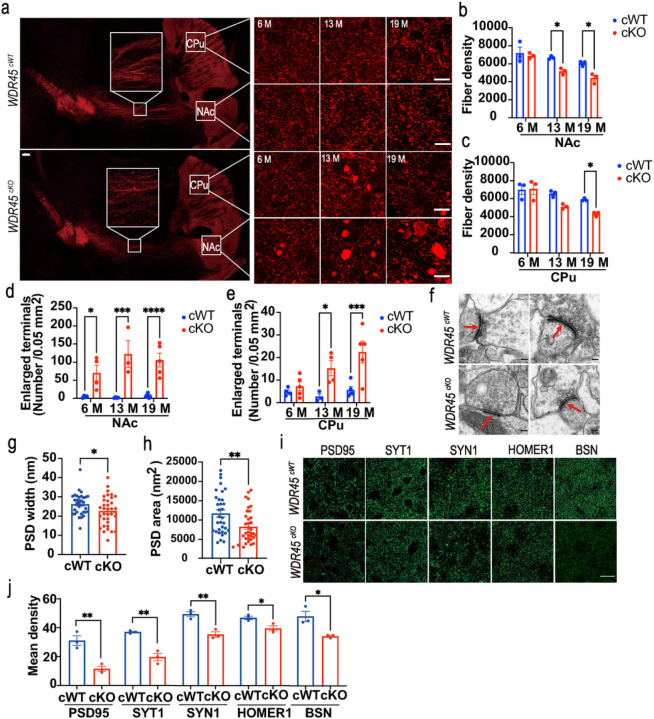
Axonal degeneration in the striatum of *WDR45*^*cKO*^ mice. a IFC staining for striatal axons, including the NAc and CPu, was performed using an antibody against TH (red) in young, middle-aged, and aged *WDR45*^*cWT*^ and *WDR45*^*cKO*^ mice. Scale bar, 200 μm. For high-magnification images: 50 μm. b, c Quantifying the fiber density in the NAc and CPu, respectively (N= 3 mice per genotype). d, e The calculation of axonal enlargements (area > 1 μm^2^) per 0.05 mm^2^ perspective in the NAc and CPu from *WDR45*^*cWT*^ and *WDR45*^*cKO*^ mice, respectively (N= 3–5 slices from 3 mice per genotype). f Representative TEM images of the observed PSD. Scale bar, 100 nm. The PSD width (g) and PSD area (h) were quantified (N=33 PSD collectively counted from 3 *WDR45*^*cWT*^ mice and 35 PSD from 3 *WDR45*^*cKO*^ mice). i IFC analysis of synapse-related proteins in the striatum of aged *WDR45*^*cWT*^ mice and *WDR45*^*cKO*^ mice. Scale bar, 20 μm. j Quantifying targeted proteins’ fluorescence intensity (N=3 slices from 3 mice per genotype). Data (b-e) were analyzed using two-way ANOVA followed by Sidak’s multiple comparisons test and Student’s t-test (g, h, j). Data are represented as the mean±SEM. *p < 0.05, **p < 0.01, ***p < 0.001, ****p < 0.0001.

In addition to the loss of DAergic neurons in the SNc, *WDR45* deletion also led to profound nerve fiber pathology in the striatum. Our longitudinal study revealed substantial changes in DAergic axonal terminals projected to the striatum of *WDR45*^*cKO*^ mice. Specifically, we observed significant axonal enlargements in the young, middle-aged, and aged *WDR45*^*cKO*^ mice, along with reduced fiber density during aging (Fig. 2a–c). It is worth noting that significantly more enlargements were accumulated in the nucleus accumbens (NAc), which receives the projection of DAergic neurons in the VTA, than in the caudate putamen (CPu) from SNc DAergic neurons, in the middle-aged and aged *WDR45*^*cKO*^ mice (Fig. 2d, e). These findings demonstrate that *WDR45*^*cKO*^ mice develop severe DAergic axonal degeneration in the striatum prior to neuronal loss and reveal the differential axonal vulnerability of DAergic neuronal subtypes in response to *WDR45* deletion.

Since large enlargements were observed in the axonal terminals, we decided to examine whether the striatal synapses were affected. To study the effect of *WDR45* deletion on excitatory synapses of DAergic projections, we conducted TEM analysis for postsynaptic density (PSD), which contributes to information processing and memory formation by changing synaptic strength in response to neural activity (27). The results showed that PSD density was significantly reduced (Fig. 2f), and PSD width and average area were significantly reduced (Fig. 2g, h). The data suggest that the synaptic structures in the striatum of *WDR45*^*cKO*^ mice have undergone alterations. Furthermore, we assessed the levels of some synaptic proteins in the striatum, including PSD95, a membrane protein of presynaptic vesicles called synaptotagmin 1 (SYT1), a synaptic vesicle protein called synapsin-1 (SYN1), postsynaptic density scaffolding protein called homer scaffold protein 1 (HOMER1), and presynaptic cytomatrix protein bassoon (BSN). We observed a significant decrease in the fluorescence density of PSD95, SYT1, SYN1, HOMER1, and BSN in the striatum of *WDR45*^*cKO*^ mice (Fig. 2i, j), indicating alterations in synaptic protein levels. These results further support that synaptic signaling transmission is disrupted in *WDR45*^*cKO*^ mice.

### Accumulation of increased fragmented tubular ER constitutes a pathological feature of swollen axons in the WDR45 ^cKO^ mice

**Figure 3:**
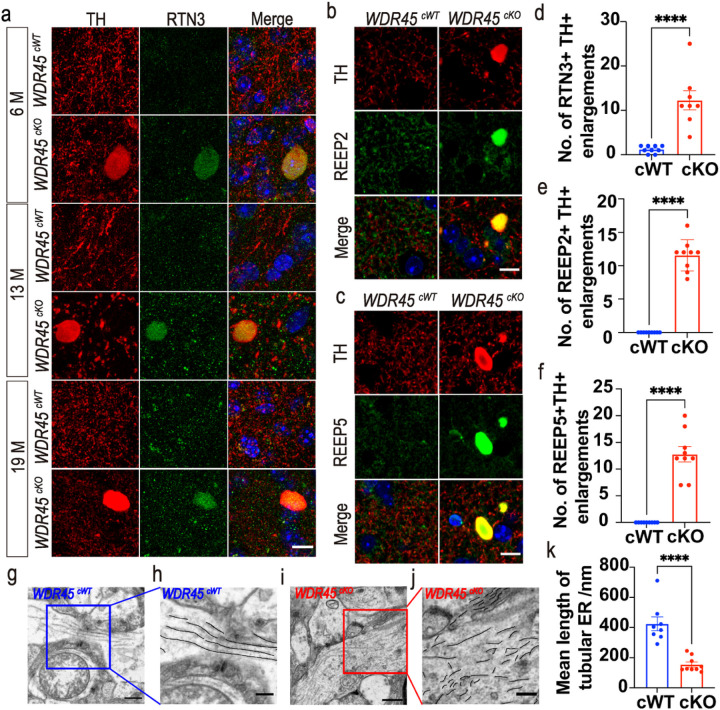
Increasing fragmented tubular ER constitutes a pathological feature of axons in *WDR45*^*cKO*^ mice. a IFC analysis for RTN3 in the NAc of *WDR45*^*cWT*^ mice and *WDR45*^*cKO*^ mice was performed using antibodies against RTN3 (green) and TH (red). The nuclei were labeled with DAPI (blue). Scale bar, 10 μm. b IFC staining for REEP2 in the NAc of aged *WDR45*^*cWT*^ mice and *WDR45*^*cKO*^ mice was performed using antibodies against REEP2 (green) and TH (red). The nuclei were labeled with DAPI (blue). Scale bar, 10 μm. c IFC staining for REEP5 in the NAc of aged *WDR45*^*cWT*^ mice and *WDR45*^*cKO*^ mice was performed using antibodies against REEP5 (green) and TH (red). The nuclei were labeled with DAPI (blue). Scale bar, 10 μm. d Analysis of RTN3- and TH-positive enlargements (N= 8–9 slices from 3 mice per genotype). e Analysis of the number of REEP2- and TH-positive enlargements (N= 9 slices from 3 mice per genotype). f Analysis of the number of REEP5- and TH-positive enlargements (N= 9 slices from 3 mice per genotype). g-j Samples from aged *WDR45*^*cWT*^ mice and *WDR45*^*cKO*^ mice were examined by TEM, and representative TEM images of observed tubular ER at the axons of the striatum are shown. The tubular ER is highlighted in black. Scale bar, 500 nm. For enlarged images, 250 nm. k The mean length of tubular ER was analyzed from aged *WDR45*^*cWT*^ mice and *WDR45*^*cKO*^ mice (N=8–9 slices from 3 mice for each genotype). Data were analyzed by using Student’s t-test. Data are represented as the mean±SEM. ****p < 0.0001.

Axonal swellings (axonal beadings, bubblings, or spheroids) are hallmarks of degenerating axons, almost universal in neurodegenerative diseases (28, 29). In our study, *WDR45* depletion in the DAergic neurons resulted in axonal swellings in the striatum. To gain insights into the molecular basis of axonal degeneration, we evaluated potential candidates by investigating their locations at the axonal enlargements. First, we examined the ER proteins Lysine-Aspartic acid-Glutamic acid-Leucine (KDEL) and climp-63 (30), the tubular ER protein ATL3, and the tubular ER-shaping proteins RTN3 and RTN4 (31, 32). RTN4, KDEL, Climp-63, and ATL3 were not observed in the axonal enlargements (Fig. S3). By contrast, RTN3 was highly concentrated at the striatal axonal enlargements in young, middle-aged, and aged *WDR45*^*cKO*^ mice (Fig. 3a, d), suggesting that RTN3 is one of the enlargement components and may contribute to the formation of axonal swellings as an early pathogenic event. Additionally, the density of RTN3-positive puncta at the striatal axons was higher in the older *WDR45*^*cWT*^ mice than in the young *WDR45*^*cWT*^ ones (Fig. 3a), indicating that aging is associated with RTN3 accumulation at the axons. As a typical tubular ER-shaping protein, the accumulation of RTN3 implies that the tubular ER shape may be affected in the striatum. We then investigated the molecular composition of axonal enlargements by determining other tubular ER-shaping proteins, REEP2 and REEP5 (33, 34). We found that REEP2 and REEP5 also colocalized with TH-positive enlargements (Fig. 3b, c, e, f), further indicating that the shape of tubular ER in the axons was disrupted upon *WDR45* depletion. These findings highlight the crucial role of ER-shaping proteins in forming axonal enlargements, providing further evidence for the importance of maintaining a normal tubular ER shape in regulating distal axonal homeostasis. The above findings prompted us to determine whether the tubular ER shape is abnormal in the *WDR45*^*cKO*^ mice. We then examined the tubular ER ultrastructure by TEM in the striatal samples from aged *WDR45*^*cWT*^ and *WDR45*^*cKO*^ mice. Compared to the normally distributed tubular ER in *WDR45*^*cWT*^ mice, a remarkably large accumulation of fragmented tubular ER was noticed in the axons of *WDR45*^*cKO*^ mice (Fig. 3g–k), supporting the notion that the fragmented tubular ER cluster is a major pathological abnormality associated with axonal degeneration in *WDR45*^*cKO*^ mice.

### Disrupted autophagic flux in the DAergic neurons may contribute to the accumulation of tubular ER in axons

**Figure 4:**
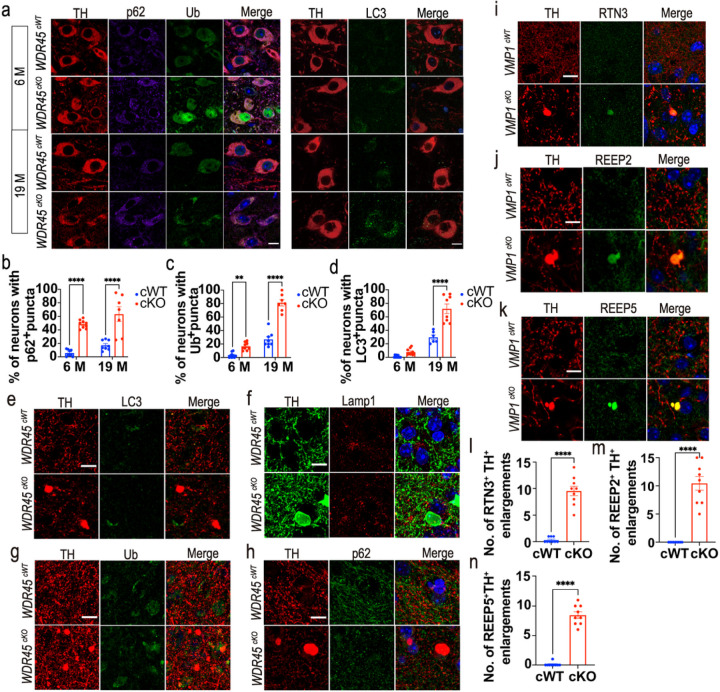
Disrupted autophagic flux in the DAergic neurons may contribute to the accumulation of tubular ER in axons. a Left panel: IFC staining for p62 (purple) and Ub (green) in the TH-positive neurons (red) of *WDR45*^*cWT*^ mice and *WDR45*^*cKO*^ mice. The nuclei were labeled with DAPI (blue). Scale bar, 10 μm. Right panel: IFC staining for LC3 (green) in the TH-positive (red) DAergic neurons. The nuclei were labeled with DAPI (blue). Scale bar, 10 μm. b The proportion of TH-positive neurons with p62 puncta (> 0.5 μm^2^) is presented (N= 3 mice per genotype). c The proportion of TH-positive neurons with Ub-positive puncta (> 0.5 μm^2^) is presented (N= 3 mice per genotype). d The proportion of TH-positive neurons with LC3-positive puncta (> 0.5 μm^2^) is presented (N= 3 mice per genotype). e-h LC3 (green), Lamp1 (red), Ub (green), and p62 (green) were detected in the NAc of aged *WDR45*^*cWT*^ mice and *WDR45*^*cKO*^ mice. The nuclei were labeled with DAPI (blue). Scale bar, 10 μm. i-k IFC staining of RTN3, REEP2, and REEP5 in the NAc of 12-month-old *VMP1*^*cWT*^ mice and *VMP1*^*cKO*^ mice was performed using antibodies against RTN3 or REEP2 or REEP5 (green) with TH (red), respectively. The nuclei were labeled with DAPI (blue). Scale bar, 10 μm. l-n Analysis of the number of RTN3- and TH-positive enlargements, REEP2- and TH-positive enlargements, and REEP5- and TH-positive enlargements, respectively (N= 9 slices from 3 mice per genotype). Data were analyzed using two-way ANOVA followed by Sidak’s multiple comparisons tests (b-d) and Student’s t-test (l-n). Data are represented as the mean±SEM. ****p < 0.0001, **p < 0.01.

To understand what contributes to the accumulation of tubular ER at axons, we first examined whether autophagic flux was disrupted in the DAergic neurons of *WDR45*^*cKO*^ *WDR45*^*cWT*^ mice, we observed distinct p62-positive puncta accumulated in the soma of DAergic neurons in young *WDR45*^*cKO*^ mice, and this accumulation was aggravated in the aged *WDR45*^*cKO*^ mice (Fig. 4a, b). Additionally, we found that Ub expression was significantly increased in the nucleus of DAergic neurons of young *WDR45*^*cKO*^ mice and in the cytoplasm of DAergic neurons of aged *WDR45*^*cKO*^ mice, of which the Ub staining was not entirely colocalized with p62-positive puncta (Fig. 4a, c). Similarly, LC3-positive puncta were also concentrated in the cell body of DAergic neurons in the *WDR45*^*cKO*^ mice (Fig. 4a, d). These data suggest that *WDR45* depletion induced an early impairment of autophagic flux in the DAergic neurons, likely triggering axonal and cell body degeneration.

To assess whether LC3-labeled autophagosomes are presented in axonal enlargements, we stained the striatal sections and found that LC3-positive puncta were absent in the TH-positive axonal enlargements (Fig. 4e), indicating that the LC3-labeled autophagosomes did not directly contribute to the formation of axonal enlargements. Furthermore, lysosome marker Lamp1 was also absent in the axonal enlargements (Fig. 4f). The autophagic substrates p62 and Ub were also not colocalized with axonal enlargements (Fig. 4g, h). Therefore, the accumulations of autophagic proteins were mainly observed in the soma but not in the axons of DAergic neurons deficient in WDR45. We speculate that disrupting autophagic flux in the DAergic neurons may lead to axonal enlargements by promoting tubular ER accumulation at axons. To test this hypothesis, we employed another mouse model with damaged autophagic flux, the *VMP1*^*cKO*^ mice that conditionally knocked out autophagic gene *VMP1* in the DAergic neurons upon TAM treatment postnatally (26). *VMP1*^*cKO*^ mice also displayed severe damage to autophagic flux and large axonal enlargements in the striatum (26). RTN3, REEP2, and REEP5 were highly accumulated at the TH-positive axonal enlargements in the striatum of 12-month-old *VMP1*^*cKO*^ mice (Fig. 4i–n), indicating that defective autophagy may induce axonal accumulation of tubular ER. Together, these results suggest that the abnormal clustering of tubular ER in axons may have pathological effects on the brain. Furthermore, our findings provide additional evidence that autophagy plays a critical role in maintaining axonal homeostasis by regulating the shape and accumulation of tubular ER.

### Exploring the proteome landscape of striatum in the WDR45 ^cKO^ mice

**Figure 5:**
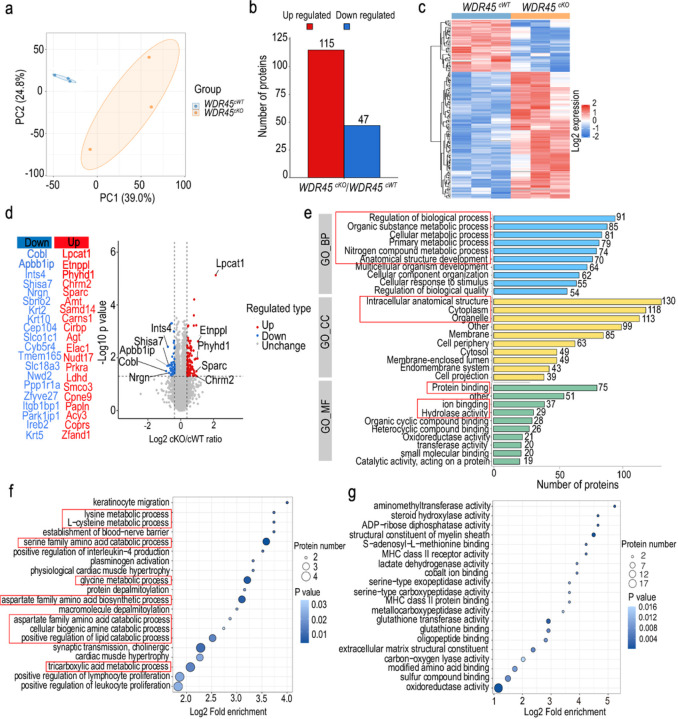
The proteome landscape of striatum in the *WDR45* ^*cKO*^ mice. a PCA score plot of the striatal proteome of aged *WDR45*^*cWT*^ and *WDR45*^*cKO*^ mice. b The statistical analysis for DEPs (fold change >1.3, *p <* 0.05). c The heatmap of the DEPs from *WDR45*^*cWT*^ mice and *WDR45*^*cKO*^ mice. d Volcano plots and top 20 up- or down-regulated DEPs organized by fold change in the striatum of *WDR45*^*cKO*^ mice vs. *WDR45*^*cWT*^ mice (DEPs marked by red and blue circles). e The top 10 CC, MF, and BP terms in GO annotation analysis. f The top 20 terms related to BP in the GO enrichment analysis. g The top 20 terms related to MF in the GO enrichment analysis.

To gain a deeper molecular understanding of how the pathological abnormalities in the DAergic axons affect the striatal cells, we dissected the striatal samples from aged *WDR45*^*cKO*^ mice and age- and sex-matched *WDR45*^*cWT*^ mice for proteomic analysis. Principal component analysis (PCA) revealed the distinct proteome profiles of *WDR45*^*cWT*^ mice and *WDR45*^*cKO*^ mice (Fig. 5a). Further proteomic analyses registered 6,290 targets, of which 162 differentially expressed proteins were identified (DEPs; > 1.3-fold change cutoff, *p <* 0.05). Among the differentially expressed proteins, 115 DEPs were up regulated, and 47 DEPs were downregulated (Fig. 5b, c, and Supplementary Table 1). The majority of the top 20 up-regulated proteins were enzymes that are involved in the regulation of lipid metabolic process, such as lysophosphatidylcholine acyltransferase 1 (Lpcat1), ethanolamine-phosphate phospho-lyase (Etnppl), abhydrolase domain containing 4, N-acyl phospholipase B (Abhd4), and phytanoyl-CoA dioxygenase domain-containing protein 1 (Phyhd1), and in the regulation of nitrogen compounds’ metabolic process, such as ElaC ribonuclease Z1 (Elac1), aminomethyltransferase (Amt), lactate dehydrogena se D (Ldhd), and cold-inducible RNA binding protein (Cirbp), as well as in regulating anatomical structure morphogenesis, like secreted protein acidic and cysteine-rich (Sparc), angiotensinogen (Agt), and the protein activator of interferon-induced protein kinase EIF2AK2 (Prkra) (Fig. 5d). The top 20 downregulated DEPs are most associated with nitrogen compounds’ metabolic process, such as complex integrator subunit 4 (Ints4), keratin 2 (Krt2), and strawberry notch homolog 2 (Sbno2), and proteins with cell morphogenesis, such as protein cordon-bleu (Cobl), amyloid β-A4 precursor protein-binding family B member 1-interacting protein (Apbb1ip) (Fig. 5d).

To support the biological significance of these DEPs, we performed Gene Ontology (GO) annotation analysis, which depicts protein functions in three categories: biological processes (BP), cellular components (CC), and molecular functions (MF). The most correlated BP of the DEPs is the regulation of the biological process, metabolic-related process, including organic substance, cellular, primary, and nitrogen compound metabolic process, and that regulation of anatomical structure development (Fig. 5e). Regarding CC, these DEPs are mostly found in the intracellular anatomical structure, cytoplasm, and organelle (Fig. 5e). The analysis results of MF show that these DEPs are primarily associated with protein binding, ion binding, and hydrolase activity (Fig. 5e). To further investigate the functions and signaling pathways of the DEPs, we performed GO enrichment analyses. The top 20 enriched BP pathways are related to amino acids’ catabolic and biosynthetic processes, positive regulation of the lipid catabolic process, protein depalmitoylation, etc. Notably, many DEPs are involved in regulating enzymatic activity, like aminomethyltransferase and steroid hydroxylase, that are enriched in the amino acid metabolic process pathway, including lysine, L-cysteine, serine family amino acids, glycine, aspartate family amino acids, and in the tricarboxylic acid metabolic process, lipid catabolic process (Fig. 5f, g).

Overall, the proteomic data indicate that most DEPs are related to lipid and amino acid metabolism, particularly catabolism. This suggests that deleting WDR45 in DAergic neurons leads to significant metabolic changes in the striatum, resulting in catabolic reactions in amino acids and lipids. The activated catabolism of these molecules may indicate an energy supply deficit in the striatum, supported by the enrichment of DEPs in the tricarboxylic acid metabolic process that produces adenosine triphosphate (ATP) for cellular energy (Fig. 5f).

### The connection of the phospholipid metabolism-related genes with the striatal pathology

**Figure 6:**
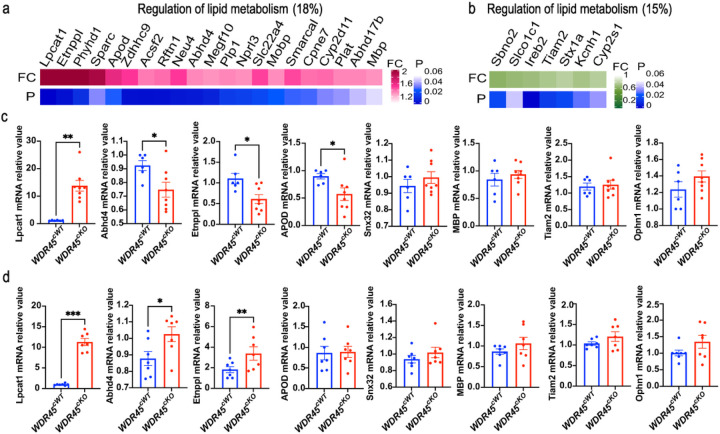
The qRT-PCR analysis for the expressions of genes encoding DEPs of regulation in lipid metabolism. GO analysis for BP displays up-regulated DEPs (pink) (a) and downregulated DEPs (green) (b) that participate in the regulation of lipid metabolism. Lower blue bars represent the magnitude of p values. Percentages indicate the fraction of each category of total up- or down-regulated DEPs. c qRT-PCR analysis for the mRNA expressions of target genes from the striatum of young *WDR45*^*cWT*^ and *WDR45*^*cKO*^ mice (n=6–8 mice per genotype). d qRT-PCR analysis for the mRNA expressions of target genes from the striatum of aged *WDR45*^*cWT*^ and *WDR45*^*cKO*^ mice (n=7–8 mice per genotype). Data were analyzed using Student’s t-test. Data are represented as the mean±SEM. *p < 0.05, **p < 0.01, ***p < 0.001.

Tubular ER dynamics are closely related to phospholipid metabolism (19). According to the proteomic data, 18% of up-regulated DEPs and 15% of down-regulated DEPs regulate lipid metabolism (Fig. 6a, b). To further understand the correlation of striatal pathology with lipids, especially with phospholipids, we analyzed those DEPs related to lipid metabolism by examining the message RNA (mRNA) level using qRT-PCR in both young and aged *WDR45*^*cKO*^ mice. The mRNA level of Lpcat1, a gene that encodes an enzyme that plays a role in phospholipid metabolism, specifically in the conversion of lysophosphatidylcholine to PC, was significantly up-regulated in the striatum of both young and aged *WDR45*^*cKO*^ mice (Fig. 6c, d), indicating that Lpcat1 is involved in the early events following the *WDR45* depletion; more importantly, the trend of change in the expression of Lpcat1 in the young and aged mice is consistent, suggesting that Lpcat1 may be one of the initiating factors for striatal axonal pathology. Additionally, we detected the expressions of other genes that participate in lipid metabolism, including Abhd4, Etnppl, apolipoprotein D (APOD), sorting nexin 32 (Snx32), and myelin basic protein (MBP), TIAM Rac1 Associated GEF 2 (Tiam2), Oligophrenin 1 (Ophn1). The results showed that the expressions of Abhd4 and Etnppl, both participate in the regulation of the phospholipid metabolic process, were significantly reduced in the striatum of young *WDR45*^*cKO*^ mice compared to that in young *WDR45*^*cWT*^ mice (0.92 vs 0.74, 1.10 vs 0.61, respectively) while markedly increased in the aged *WDR45*^*cKO*^ mice (0.88 vs 1.02, 1.83 vs 3.38, respectively), indicating that the expression of these two genes is simultaneously affected by aging or aging-related processes (Fig. 6c, d). The expression of APOD was significantly downregulated in the young *WDR45*^*cKO*^ mice but showed no apparent alteration in the aged *WDR45*^*cKO*^ mice (Fig. 6c, d). Snx32, MBP, Tiam2, and Ophn1 expression showed an up-regulated trend in the young and aged *WDR45*^*cKO*^ mice (Fig. 6c, d). In summary, our findings emphasize the role of phospholipid biosynthesis and catabolism-regulating molecules in the striatal pathology resulting from WDR45 dysfunction.

## Discussion

**Figure 7:**
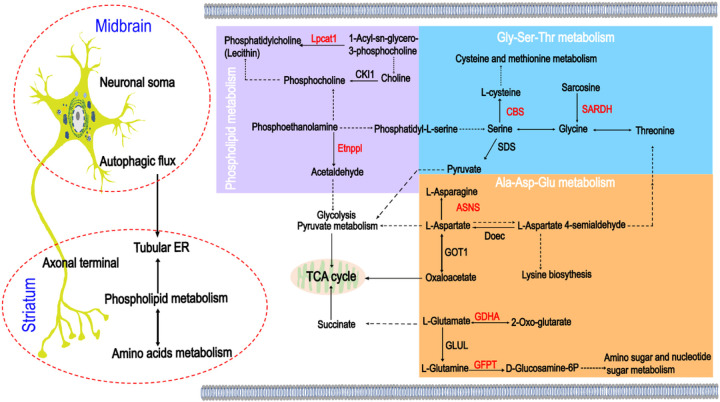
The potential interactions of autophagy, tubular ER, and metabolic processes in the axonal pathology of WDR45-deficiency-induced DAergic neurodegeneration. Autophagy is essential for maintaining axonal homeostasis by controlling tubular ER. Phospholipid metabolism interacts with amino acid metabolism and potentially regulates tubular ER. These complex interactions jointly regulate axonal homeostasis. The right panel shows the perturbed pathways in phospholipid metabolism, Gly-Ser-Thr metabolism, and Ala- Asp-Glu metabolism, as well as the interactions among them. The texts marked in red represent the up-regulated DEPs. Black arrows show single-step enzyme catalysis. The black dashed arrows indicate multi-step enzyme catalysis. Abbreviations: Gly, glycine; Ser, serine; Thr, threonine; Ala, alanine; Asp, aspartate; Glu, glutamate; Lpcat1, lysophosphatidylcholine acyltransferase 1; CKI1, choline kinase1; Etnppl, ethanolamine-phosphate phospho-lyase; CBS, cystathionine beta-synthase; SDS, L-serine/L-threonine ammonia-lyase; SARDH, sarcosine dehydrogenase; ASNS, asparagine synthase; Doec, aspartate-semialdehyde dehydrogenase; GOT1, aspartate aminotransferase1; GDHA, glutamate dehydrogenase; GFPT, glutamine-fructose-6-phosphate transaminase; GLUL, glutamine synthetase.

In this study, we generated and characterized *WDR45*^*cKO*^ mice, which exhibited neurodegeneration of midbrain DAergic neurons, particularly axonal degeneration. We observed the accumulation of abundant fragmented tubular ER in axonal enlargements, impaired autophagic flux in cell bodies, and alterations in DEPs that regulate phospholipid biosynthesis and catabolism. These findings provide a possible molecular mechanism for axonal degeneration in DAergic neurodegeneration induced by WDR45 deficiency (Fig. 7).

Several neurodegenerative diseases, including Alzheimer’s disease, Parkinson’s disease, and amyotrophic lateral sclerosis, exhibit apparent defects in axonal transport (35, 36). These defects manifest as axonal swellings or spheroids with an aberrant accumulation of axonal cargoes, cytoskeletal proteins, and lipids (37). Prominent axonopathy is induced by the overexpression of pathological proteins involved in several neurodegenerative diseases (37, 38). Recent studies have shown that autophagy plays a vital role in axonal homeostasis. Inoue et al. demonstrated that autophagy could regulate mature DAergic axon terminal morphology (39). They found enlarged axonal terminals selectively in the mature ATG7-deficient DAergic neurons (39). Similar axonal enlargements were found in the *WDR45*^*cKO*^ mice, VMP1-deficient DAergic neurons (26), and Perry syndrome-associated p150^Glued^-deficient DAergic neurons (40). However, the underlying mechanism of how autophagy regulates axonal morphology is still unclear. One study suggests autophagy regulates axonal transmission by controlling axonal ER (20). They found that when ATG5 was deleted from hippocampal neurons, tubular ER was selectively accumulated in axons, followed by ryanodine receptors relative to ER stores in axons, resulting in aberrant calcium release and neurotransmission (20). Moreover, Wan et al. found that WDR45 contributed to neurodegeneration by regulating ER stress and ER quality control, suppressing ER stress, or activating autophagy through mTOR inhibition, alleviating cell death (4). Their observations emphasize the regulation of WDR45 in ER homeostasis through the macroautophagy machinery, which aligns with our hypothesis that axonal swellings in *WDR45*^*cKO*^ mice originate from axonal ER accumulation and that autophagy is responsible for the axonal ER accumulation. Tubular ER is responsible for lipid biogenesis and calcium signaling and provides contact sites for other organelles involved in axonal pathology (19, 41). In our study, the morphology of tubular ER in axons is impacted when WDR45 is deficient. Tubular ER becomes fragmented and accumulates in the axons, ultimately promoting axonal enlargement formation. Conversely, sheet ER, which represents rough ER, does not participate in axonal pathology because it is mainly responsible for protein synthesis, processing, and sorting in the soma (41), as confirmed by our finding that the sheet ER markers, KDEL, and climp63, do not colocalize with axonal enlargements in *WDR45*^*cKO*^ mice.

Our results indicate that the morphology of tubular ER is important in maintaining axonal homeostasis, which is supported by the proteomic data showing the strong response of phospholipid metabolism in the striatum to WDR45 depletion. The biosynthesis and catabolism of phospholipids play a fundamental role in the morphology and composition of the ER (16). Lpcat1, an important ER-resident enzyme that catalyzes PC biosynthesis, is substantially up-regulated in the striatum of *WDR45*^*cKO*^ mice, indicating that WDR45 directly or indirectly inhibits Lpcat1 expression and through which WDR45 may participate in the regulation of axonal ER morphology. Previous studies have emphasized the important role of Lpcat1 in lung cancers (42), and recent studies have shown that Lpcat1 also plays a role in neurological diseases. Lpcat1 regulated α-synuclein (αSyn) pathology and cytotoxicity (43). Suppression of Lpcat1 reduces αSyn accumulations and toxicity, and overexpression of Lpcat1 promotes phosphorylated S129 αSyn positive aggregation (43). A phospholipid product of Lpcat1 enzymatic activity, 1,2-dipalmitoyl-sn-glycero-3-phosphocholine, similarly promotes neuronal αSyn pre-formed fibril-seeded aggregation (43). The relationship between axonal degeneration induced by WDR45 depletion and Lpcat1 expression and associated phospholipid products is an interesting area for future research.

Recent studies indicate phospholipids, and their corresponding compounds are essential for autophagy regulation. The de novo formation of autophagosomes originates from the biosynthesis of phospholipids (44), and the loss of PC biosynthesis compromises the closure of the autophagic membrane and autophagic flux (45). Mitophagy can be inhibited by changing the LC3 residues that carry the cardiolipin-interaction sites. Cardiolipin is an analogy to phospholipid found in the inner mitochondrial membrane. It is an elimination signal for mitophagy in neural cells (46). The mutation of Spns1, a protein that acts as a proton-dependent lysophosphatidylcholine (LPC) and lysophosphatidylethanolamine (LPE) transporter, will lead to lysosomal accumulation of LPC and LPE with pathological consequences on lysosomal function (47). Despite these studies supporting the regulation of phospholipids on autophagy, there is currently no report that WDR45 regulates the autophagy process through phospholipids. Our study demonstrates that the deletion of WDR45 causes axonal swellings and fragmented tubular ER accumulation in the swollen axons. To determine the underlying pathways and molecular mechanisms regulating the axonal pathology, we performed proteomic analysis of the striatum from *WDR45*^*cKO*^ mice compared with its littermate *WDR45*^*cWT*^ mice. We found significant changes in the expression of phospholipid metabolism-related regulatory proteins, indicating that phospholipid metabolism may contribute to axonal degeneration. To further clarify the regulation of lipid metabolism on tubular ER and its relationship with autophagy will significantly improve the mechanistic understanding of axonal degeneration in many neurodegenerative diseases.

Additionally, we screened several pathways by GO enrichment and found that the most activated pathways include the phospholipid metabolic process, amino acids metabolic process, and tricarboxylic acid metabolic process. The defects in these pathways may cause major disturbances in the catabolism of amino acids, lipid catabolism, and tricarboxylic acid metabolism, all related to energy supply. The amino acid metabolism interacts with lipid metabolism in regulating neuronal excitability and survival (48), and these metabolic disturbances are closely related to neurodegeneration (49, 50). Due to their anatomical structure, axons are more sensitive to endogenous and exogenous stimuli, such as energy shortages. Mitochondria supply the energy of neurons through oxidative phosphorylation reactions, but given the elongated anatomical structure of the axons, the local ATP supply of the axons is more critical for their survival. Mitochondria are anchored at distal axons and synapses ideally as local energy sources by generating ATP through oxidative phosphorylation (51). Regulating the trafficking and anchoring status of axonal mitochondria ensures that metabolical areas are constantly supplied with ATP. Several pathways and molecules are crucial in regulating mitochondrial transport to meet axonal energy supply and facilitating axonal regeneration, such as AMP-activated protein kinase-p21-activated kinase energy signaling pathway, myosin VI (52), AKT-P21-activated kinase 5 axis (53), syntaphilin (54). Combined with the DEPs in our proteomic data, these pathways provide a clue to study the mechanism of energy supply damage in the axonal degeneration of neurodegenerative diseases.

## Conclusions

In conclusion, our research provides insights into the pathological mechanisms underlying WDR45 deficiency-induced axonal degeneration. Our findings suggest that this degeneration involves abnormal phospholipid metabolism and damaged autophagy. While this study provides a molecular basis for axonal degeneration in BPAN and other neurodegenerative diseases, further mechanistic investigations are necessary to fully understand the complex relationship between tubular ER, autophagy, and phospholipid metabolism in axonal degeneration. The elucidation of such a relationship will facilitate the development of more effective strategies for preventing or reversing axonal degeneration in the context of WDR45 deficiency and related disorders.

## Figures and Tables

**Table 1:** Antibodies used in this study.

Target	Species	Application	Dilution	Company	Cat. no
**TH**	Mouse	IFC	1:1000	Sigma	T1299
**TH**	Rabbit	IFC	1:1000	Millipore	AB152
**WDR45**	Rabbit	IFC	1:500	Novus	NBP3–04699
**LC3β**	Rabbit	IFC	1:400	Novus	NB100–2220
**LC3β**	Mouse	IFC	1:500	CST	83560s
**P62**	Rabbit	IFC	1:400	Abeam	Ab109012
**Lamp1**	Rat	IFC	1:500	Abeam	Ab25245
**MLKL (phospho S345)**	Rabbit	IFC	1:400	Abeam	Abl96436
**Ubiquitin**	Mouse	IFC	1:200	SANTACRUZE	Sc8017
**RTN3**	Rabbit	IFC	1:1000	Millipore	ABN1723
**RTN2**	Rabbit	IFC	1:1000	Millipore	ABN1723
**REEP5**	Rabbit	IFC	1:500	Proteintech	14643–1-AP
**Phospho-RIP3 (Thr231/Ser232)**	Rabbit	IFC	1:400	CST	91702
**KDEL**	Rabbit	IFC	1:250	Abeam	Abl76333
**RTN4**	Mouse	IFC	1:300	SANTA	Sc-271878
**Climp-63**	Mouse	IFC	1:300	SANTA	Sc-393544
**Calnexin**	Mouse	IFC	1:300	SANTA	Sc-23954
**Lamp1**	Rat	IFC	1:500	Proteintech	65050–1-Ig
**PSD95**	Rabbit	IFC	1:500	Synaptic system	N3783
**Synapsin1**	Rabbit	IFC	1:500	Synaptic system	106103
**Synaptotagmin 1**	Mouse	IFC	1:500	Synaptic system	105011
**H0MER1**	Rabbit	IFC	1:500	Synaptic system	160003
**Bassoon**	Chicken	IFC	1:500	Synaptic system	141016

**Table 2:** Primers used for qRT-PCR.

Primer	5’-F	5’-R
**Lpcat1**	GGCTCCTGTTCGCTGCTTT	TTCACAGCTACACGGTGGAAG
**Snx32**	GCTGGAAATGAGAGTAAGCCTT	GGTGTCATGTAGCCAGATGAACT
**Abhd4**	GGCACAGTTTGGGAGGATTCC	ACTAGGGTCAGTTGGTCGTAG
**Etnppl**	AGAGGGAGGAACATTCATTGACT	GGCTCGCATTATTTTGATGGGA
**MBP**	GGCGGTGACAGACTCCAAG	GAAGCTCGTCGGACTCTGAG
**APOD**	TCACCACAGCCAAAGGACAAA	CGTTCTCCATCAGCGAGTAGT
**Tiam2**	ACATGGTTGGACTCATGGGAG	TGGTGCCCTTTGAGACTTTACA
**Ophn1**	ACCCCTGGAAACTTTTCGGAA	TCTGCCTCTAGTAGCTGAGATTC
**GAPDH**	AGGTCGGTGTGAACGGATTTG	TGTAGACCATGTAGTTGAGGTCA

## Data Availability

All data generated in this study are included in this published article. Raw datasets during the current study are available from the corresponding author upon request.

## Abbreviations

ATG5: autophagy gene 5
ATL: atlastin
Amt: aminomethyltransferase
ATP: adenosine triphosphate
αSyn: αSynuclien
Abhd4: abhydrolase domain containing 4, N-acyl phospholipase B
APOD: apolipoprotein D
Agt: angiotensinogen
Apbb1ip: amyloid β-A4 precursor protein-binding family B member 1-interacting protein
αSyn: αSynuclien
BPAN: β-propeller protein-associated neurodegeneration
BSN: presynaptic cytomatrix protein bassoon
BP: biological processes
CPu: caudate putamen
CDP-DAG: cytidine diphosphate-triacylglycerol
Cirbp: cold inducible RNA binding protein
Cobl: protein cordon-bleu
CC: cellular components
DAergic: dopaminergic
DEPs: differentially expressed proteins
ER: endoplasmic reticulum
Etnppl: ethanolamine-phosphate phospho-lyase
Elac1: elaC ribonuclease Z1
GO: gene ontology
HOMER1: homer scaffold protein 1
Ints4: integrator complex subunit 4
IFC: immunofluorescence
KDEL: lysine-aspartic acid-glutamic acid-leucine
Krt2: keratin 2
DAergic: midbrain dopaminergic
Ldhd: lactate dehydrogenase D
Lpcat1: lysophosphatidylcholine acyltransferase 1
MLKL: mixed lineage kinase-like
MBP: myelin basic protein
MF: molecular functions
NAc: nucleus accumbens
Ophn1: Oligophrenin 1
PSD: postsynaptic density
PC: phosphatidylcholine
PIS: phosphatidylinositol synthase
PCA: principal component analysis
Phyhd1: phytanoyl-CoA dioxygenase domain-containing protein 1
Prkra: protein activator of interferon induced protein kinase EIF2AK2
RIPK3: receptor-interacting protein kinase-3
RTN: reticulon
REEP: receptor accessory protein
SNc: substantia nigra pars compacta
SN: substantia nigra
SYT1: synaptotagmin 1
Sparc: secreted protein acidic and cysteine rich
Sbno2: strawberry notch homolog 2
Snx32: sorting nexin 32
TAM: tamoxifen
TH: tyrosine hydroxylase
Tiam: TIAM Rac1 Associated GEF 2
SYN1: synaptic vesicle protein synapsin-1
VTA: ventral tegmental area
WDR45: WD repeat domain 45
